# Coupling and metabolic analysis of urbanization and environment between two resource-based cities in North China

**DOI:** 10.7717/peerj.6869

**Published:** 2019-05-02

**Authors:** Hao Chen, Linyu Xu, Qingqing Cao, Miansong Huang, Minghua Song, Quan Quan, Jian Liu

**Affiliations:** 1 Institute of Environmental Research, Shandong University, Qingdao, China; 2 State Key Laboratory of Environmental Simulation and Pollution Control, School of Environment, Beijing Normal University, Beijing, China; 3 School of Architecture and Urban Planning, Shandong Jianzhu University, Jinan, China; 4 Ningxia Capital Sponge City Construction & Development Co., Ltd, Guyuan, China; 5 Key Laboratory of Ecosystem Network Observation and Modeling, Institute of Geographic Sciences and Natural Resources Research, Chinese Academy of Sciences, Beijing, China; 6 State Key Laboratory of Eco-hydraulics in Northwest Arid Region of China, Xi’an University of Technology, Xi’an, China

**Keywords:** Urbanization, Metabolism, Coupling, Emergy, Environmental loading ratio

## Abstract

**Background:**

The complex relationship between urbanization and environment in resource-based cities is of increasing concern.

**Methods:**

As typical examples of rapid economic growth, obvious urbanization, and successful transformed production models, the cities of Dongying and Binzhou in Yellow River Delta High-tech Economic Zone were chosen for research. First, this study examines the coupling relationship between urbanization and the environment over the last seventeen years using the coupling degree model. Second, the emergy analysis method is used to further study the energy metabolism and environmental load in the two cities to reveal these couplings.

**Results:**

Dongying and Binzhou were well-coupled and the coupling coordination degree was in the stage of mild coordination coupling showing an upward trend. The total metabolic energy of the two cities increased yearly from 2000 to 2016, and the emergy extroversion ratio data showed the cities’ dependence on external elements such as continuously increased imported resources. The total emergy used in the two cities showed an upward trend during 2000 and 2016, while the emergy per capita consumption increased significantly, suggesting that the society’s energy efficiency improved. During the same period, the environmental loading ratio increased gradually, and the elements causing the environmental load shifted from internal to external.

**Discussion:**

The study shows that the factors of environmental load in developing cities are gradually shifting from internal to external, which is vital to understanding the impact of urban transformation and upgrading of resource-based cities on the environment.

## Introduction

Propelled by the progress of technology and society, urban areas have become the center of human life, production, and cultural exchange. Statistically, 54% of the world’s population was concentrated in urban areas in 2016, and this proportion will increase in the future. However, the national economic development is restricted by the level of cities’ urbanization (Cohen, 2008), especially the process of urbanization in China is faster than that in other countries (Fang & Yu, 2016). As the main development model of new urbanization, urban agglomerations can provide the necessary support for development (Liu, Xu & Luo, 2014). Nevertheless, this inevitably entails occupation of ecological land and increased consumption leads to conflicts with the environment during the urbanization process (Pascual & Abollo, 2003). Therefore, to describe and study the conflicts between urbanization and environment, researchers draw on the coupling concept in physics and define the interactive coercion as the coupling between urbanization and environment (Huang & Fang, 2003). The coupling theory, which includes coupling degree and coupling coordination degree, can clearly reflect the degree of interaction and mutual influence between systems and judge whether the systems are harmoniously developed (Gao et al., 2012; Dang et al., 2015). Coupling degree indicates the strength of interaction between urbanization and environment, while the coupling coordination degree indicates whether urbanization and environment coordinate. In particular, the degree of coupling coordination is evidently affected by the level of economic activity ( Li et al., 2012).

In the process of urbanization, the coupling relationship between urbanization and regional environment is reflected in not only local coupling between different internal factors, but also external coupling between urban regions and external factors (Fang & Ren, 2017). The local coupling process of urbanization and environment provides the necessary resource base for the development of urban regions, while external factors in the external coupling process further provide materials, energy, labor, and investment (Liu et al., 2015; Sun & Architecture, 2016). Changes in the proportion of internal and external resources lead to constant changes in the coupling relationship between urbanization and regional environment (Wang, Fang & Wang, 2015). In the coupling procedure, the environment provides the necessary resource guarantee for the process of urbanization, which causes problems such as land loss, over-population, and pollution of the ecosystem (Liu et al., 2015). This is considered a complex metabolic process between internal and external factors in urban areas (Niza, Rosado & Ferrão, 2009), where the coupling relationship changes constantly. Therefore, to explore the trend and evolutionary mechanism of interaction between urbanization and environment, understand the coupling relationship between urbanization and environment, and understand its impact on internal and external environments in developing countries, it is necessary to comprehensively study the coupling relationship and metabolic process occurring between urbanization and environment. Although many studies have examined the coupling of urbanization and environment in the last few decades ( Li et al., 2012; Liu, Xu & Luo, 2014; Chai, Wang & Zhang, 2017), detailed research on the metabolic process between them based on coupling, especially the study of internal and external metabolic factors, is relatively scarce.

Metabolism can be analyzed using two different approaches, material flow and energy flow, which reflect the material and energy conversion process between interactive urban systems and ecosystems (Odum, 1983; Zhang, Yang & Chen, 2007). In terms of physical quality, material flow analysis determines the flow characteristics and the conversion efficiencies of urban power sources, materials, and energy in terms of conversion from raw materials to products and waste, usually in tons (Kennedy et al., 2015). However, due to differences in the physical properties of materials, the conversion process of different substances cannot be easily incorporated into the material flow analysis, which explains the lack of economic, social, and demographic factors in urban and ecological systems (Wang, Fang & Wang, 2015). In contrast, a method called emergy analysis, which was proposed by Odum in the 1980s (Odum, 1983; Odum, 1988), enables the analysis of energy flows, logistics, and other ecological flows of various ecosystems or eco-economic systems that are difficult to measure and uniformly convert into a unified unit, such as renewable resources, non-renewable resources, goods, services, and even information and education. The energy values of various resources, products, and services originate directly or indirectly from solar energy. Solar emergy is often used to measure the emergy of a certain element (Odum, 1983; Zhang, Yang & Chen, 2007). Emergy analysis results reflect the local coupling and remote coupling of resources, energy consumption intensity, and output efficiency with the environment during the urbanization of urban agglomerations. The emergy of all products and services is based on solar energy (Brown & Ulgiati, 2004).

In this study, the cities of Dongying and Binzhou are selected as the research areas, the urbanization process here is relatively fast, while resources and the environment are under great pressure. These two cities are transforming from resource-based cities to comprehensive cities and therefore they are suitable for studying the law of urban development. Research on energy metabolism was conducted in these urban areas based on the coupled urbanization and environment. By studying the data on 17 consecutive years, we combined the coupling theory calculation with the regional emergy analysis, enabling us to gain intuitive and specific insights into the relationship between urbanization and environment. This study has three main objectives. First, we try to identify the changing trend of the interaction between urbanization and environment by calculating the coupling degree and coupling coordination degree in Dongying and Binzhou from 2000 to 2016. Second, we use emergy analysis to analyze the metabolic intensity and efficiency between urbanization and environment. Third, we use emergy analysis to study the environmental load caused by internal and external elements on the environment in the process of urbanization.

## Method

### Study area

**Figure 1:**
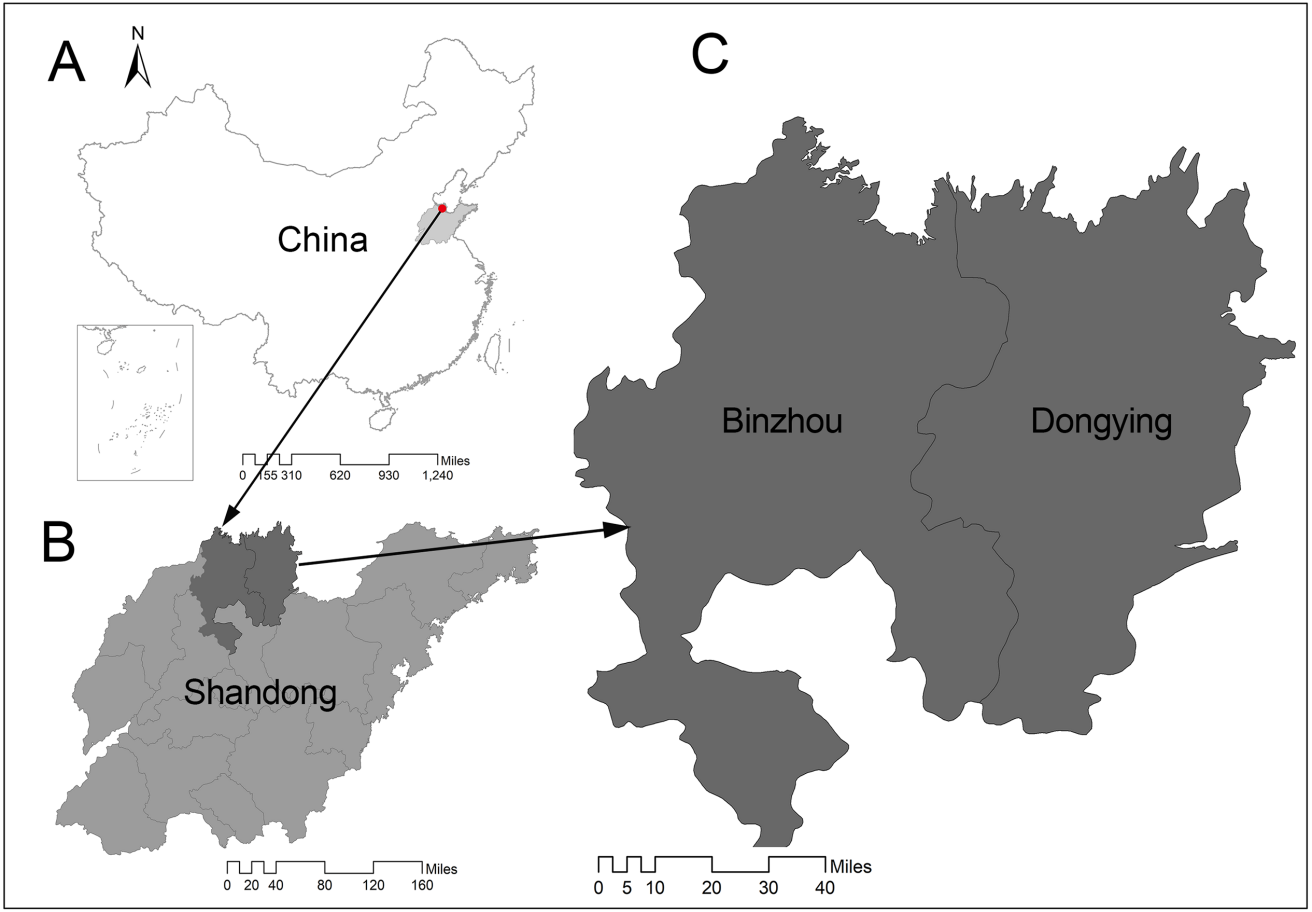
Study area. (A) Location of Shandong Province in China. (B) Dongying and Binzhou in Shandong Province. (C) The area of Dongying and Binzhou.

Dongying and Binzhou are two core cities in the Yellow River Delta High-Efficiency Ecological Economic Zone in Shandong Province, Northern China (Fig. 1).

Binzhou (36°41′∼38°16′E, 117°15′∼118°37′N) and Dongying (36°55′∼38°10′E, 118°0′∼119°10′N) are located in the northern part of Shandong Province on the south bank of the Bohai gulf in the center of the Yellow River Delta. Both cities have typical temperate monsoon climate, Binzhou has a jurisdiction of 9,453 km^2^ and population of 3.8 million, and Dongying has a jurisdiction of 8,243 km^2^ and resident population of 2.13 million. Dongying and Binzhou are rich in mineral resources and the major producing areas of Shengli Oilfield (Shandong Statistics Bureau, 2017). Dongying and Binzhou were considered as resource-based cities, due to the excessive exploitation and construction of resources has had a serious impact on the local environment load, the government has gradually taken steps to protect the environment through a series of measures such as industrial restructuring and strict implementation of energy conservation and emission reduction policies. Consequently, the two cities are transforming into comprehensive cities.

### Determination of indicators

The calculation of the coupling degree includes 25 indicators of social, economic, and environment factors in the process of urbanization (Jaeger et al., 2010) (Table 1). The selected indicators need to meet the following conditions: They must be among the most cited indicators, and they must include the necessary components of the socio-economic-ecological subsystem. The emergy analysis method typically includes 29 widely-used indicators (Higgins, 2003; Fang & Ren, 2017), including the internal and external influence factors of the coupling of urbanization and environment (Table 2).

**Table 1:** Index system of socio-economic-ecological environment factors in the process of urbanization.

Sub-system		Index & Direction	Effect
The integration value of Economic (E)	Economic structure	Secondary industry proportion of GDP (%)	+
		Tertiary industry proportion of GDP (%)	+
		Import and export trade proportion of GDP (%)	+
	Economic efficiency	Per capita annual income	+
		GDP per capita (yuan/person)	+
		Per capita industrial output value (yuan/person)	+
The integration value of Society (S)	Population and land	Urban population	+
		Per capita road area (sq.m/ person)	+
		Urban population density (person/Km^2^)	+
		Built-up area (square kilometers)	+
	Demographics	Non-agricultural population as a percentage of total population (%)	+
		Number of doctors per 10,000 people	+
		Number of college graduates per 10,000 people	+
		Percentage of employees in the tertiary industry	+
The integration value of Ecology (E)	Environmental pressure	Industrial wastewater discharge (t)	-
		Sulfur dioxide emissions (t)	-
		Industrial waste production (t)	-
	Environmental status	Per capita cultivated area (sq.m /person)	+
		Per capita land area (sq.m / person)	+
		Per capita water supply (t/person)	+
		Per capita green area (m^2^/person)	+
		Green area coverage in built-up areas	+
	Environmental response	Proportion of industrial wastewater that meets emission standards	+
		Comprehensive utilization ratio of industrial solid waste	+
		Environmental protection investment as a percentage of GDP (%)	+

**Notes.**

a “+” represents positive effect. “-” represents negative effect.

**Table 2:** Index system and transformity of the elements affecting the Dongying-Binzhou urban area.

Type	Item	Unit	Transformity (Sej/unit)	Reference
Renewable resources	Solar	J	1	Odum (1996)
	Rainfall (chemical)	J	3.05E+04	Odum (2000)
	Rainfall (potential)	J	4.70E+04	Odum (2000)
	Wind energy	J	2.45E+03	Odum (2000)
	Wave energy	J	5.10E+04	Odum (2000)
	Tidal energy	J	7.39E+04	Odum (1996) and Odum (2000)
	River (chemical)	J	8.10E+04	Odum (2000)
	River (potential)	J	4.70E+04	Odum (2000)
	Earth cycle	J	5.80E+04	Odum (2000)
	Livestock products	J	3.36E+06	Brown & Herendeen (1996)
	NPP	J	3.36E+05	Wang et al. (2016)
	Aquatic products	J	3.36E+06	Brown & Mcclanahan (1992)
Non-renewable resources	Topsoil loss	J	7.40E+04	Brown & Bardi (2001)
	Soil loss	J	1.68E+09	Odum (1996)
	Raw coal	J	6.72E+04	Odum (1996)
	Natural gas	J	5.88E+04	Romitelli (2000)
	Crude oil	J	5.04E+04	Odum (1996)
	Gasoline	J	1.86E+05	Romitelli (2000)
	Diesel	J	1.86E+05	Odum (1996)
	Fuel oil	J	6.25E+04	Odum (1996)
	Electricity	J	1.60E+05	Brandt-Williams (2001)
Waste	Water	gallon	8.77E+11	Nelson et al. (2001)
	Solid	J	1.80E+06	Jiang et al. (2009)
Input	Foreign investment	$	7.99E+12	Jiang et al. (2009)
	Domestic input	J	6.72E+04	Jiang et al. (2009)
	Imported goods and services	$	7.99E+12	Jiang et al. (2009)
	Travel	$	3.81E+12	Odum (1996)
Output	Investment output	$	9.71E+12	Odum (2000)
	Exported goods and services	$	9.71E+12	Odum (2000)

**Notes.**

a NPP: Net Primary Production.

b J: The unit of joule.

c $: The unit of dollar.

### Coupling degree model

The coupling degree model is commonly used to study the coupling between urbanization and environment (Huang & Fang, 2003). The coupling degree model divides the indicators into two systems: the socio-economic system contains 14 indicators, and the environment system contains 11 indicators (Table 1). The relevant data of the indicators are standardized to reduce magnitude and interference in the positive and negative directions to make the assessment of indicators more scientific and effective. The standardized steps include the following:

Positive index (larger value for a useful parameter):

(1) \begin{eqnarray*}{X}_{ij}= \frac{{x}_{ij}-{x}_{ijmin}}{{x}_{ijmax}-{x}_{ijmin}} .\end{eqnarray*}

Negative index (smaller value for a useful parameter):

(2) \begin{eqnarray*}{X}_{ij}= \frac{{x}_{ijmax}-{x}_{ij}}{{x}_{ijmax}-{x}_{ijmin}} \end{eqnarray*}

where *X*_*ij*_ represents the standardized value, *x*_*ij*_ represents the initial value, *x*_*ijmax*_ represents the maximum value of the system before normalization, and *x*_*ijmin*_ represents the minimum value before the system is normalized; *i*=1,2,3……; *m*; *j*=1,2,3……; *n*.

In this study, the entropy method is used to determine the weight of the indicators (Sakata & Sato, 1990). This method determines the utility value of the indicator by evaluating the intrinsic information of the indicator, and to avoid the subjective deviation to a certain extent. The entropy method is one of the preferred methods to analyze the characteristics of the coupling relationship between urbanization and environment. The steps to determine the index weight by the entropy method are as follows:

Calculate the proportion *u*_*ij*_:

(3) \begin{eqnarray*}{u}_{ij}= \frac{{x}_{ij}}{\sum _{i=1}^{n}{x}_{ij}} .\end{eqnarray*}

Calculate the entropy *h*:

(4) \begin{eqnarray*}{h}_{j}=-k\sum _{i=1}^{n}({u}_{ij}\ln \nolimits {u}_{ij}),k= \frac{1}{\ln \nolimits n} .\end{eqnarray*}

Calculate the difference rate *a*_*j*_:

(5) \begin{eqnarray*}{a}_{j}=1-{h}_{j}.\end{eqnarray*}

Calculate the entropy weight coefficient *w*_*j*_:

(6) \begin{eqnarray*}{w}_{j}= \frac{{a}_{j}}{\sum _{i=1}^{n}{a}_{j}} .\end{eqnarray*}

Calculate the contribution of the socio-economic system and environment system separately:

(7) \begin{eqnarray*}& & f(\mathrm{x})=\sum _{i=1}^{n}{w}_{j}{x}_{ij}\end{eqnarray*}

(8) \begin{eqnarray*}& & f(\mathrm{y})=\sum _{i=1}^{n}{w}_{j}{y}_{ij}.\end{eqnarray*}

Based on the coupling degree model, we calculated the coupling degree and coupling coordination degree between urbanization and the environment.

Coupling model of urbanization and the environment:

(9) \begin{eqnarray*}C={ \left\{ \frac{f \left( x \right) \cdot f(y)}{{ \left[ \frac{1}{2} \left( f \left( x \right) +f \left( y \right) \right) \right] }^{2}} \right\} }^{ \frac{1}{2} }\end{eqnarray*}

where *C* (0 ≤*C* ≤1) represents the coupling degree. When *C* ≤ 0.30, the coupling degree is extremely imbalanced and the relationship between urbanization and the ecological environment is deteriorating; when 0.30 < *C* ≤ 0.50, the coupling degree is moderately imbalanced; when 0.50 < *C* ≤ 0.80, the coupling degree is mildly coupled; and when *C* > 0.80, the coupling degree is well coupled. Generally, the reference interval is determined by the degree of urbanization (Hou et al., 2014).

However, in some cases, it is difficult to reflect the synergy between regional urbanization and environment in the coupling degree. Particularly, in comparative studies of two or more cities , it is difficult to reveal the degree of coordination between the two systems of urban socio-economic and environment (Liu, Li & Song, 2005); although the coupling degree of the two cities may be similar, it is possible that the coupling coordination degree of one city is high and the other is low. Therefore, the coupling coordination degree is necessary to study the degree of coordination between regional urbanization and environment (Liu et al., 2011; Liu, Xu & Luo, 2014; Zi, 2015). Its calculation formula is as follows:

(10) \begin{eqnarray*} \left\{ \begin{array}{@{}l@{}} \displaystyle D={ \left( C\times T \right) }^{ \frac{1}{2} } \\ \displaystyle T=a{U}_{1}+b{U}_{2} \end{array} \right. \end{eqnarray*}

where *D* (0 ≤ *D* ≤1) denotes the coupling coordination degree. The coupling coordination rank also has four levels: extremely imbalanced (*D* ≤ 0.30), moderately imbalanced (0.30<*D* ≤ 0.50), mildly coordinated (0.50<*D* ≤ 0.80), and well coordinated (*D* > 0.80). *T* is the overall effect of urbanization and environment. *U*_1_ and *U*_2_ are the adjusted evaluation indices for the urbanized and environmental subsystems; *a* and *b* (*a* + *b* = 1) represent the contributions of urbanization and environment, respectively, because the urbanized and environmental subsystems are equally important. Then, *a* and *b* are set as 0.5.

### Emergy analysis and calculation

Based on the coupling between urbanization and environment, a socio-economic-ecological composite urban ecosystem theory was proposed to quantitatively analyze the economic flow, material flow, and energy flow in cities (Ma, 1981; Wang et al., 2011a; Wang et al., 2011b). At the same time, studying the metabolism of socio-economic-ecological composite urban ecosystems is the main research method for studying the urbanization process (Kennedy, Pincetl & Bunje, 2011). Urban metabolism is a process in which cities invest resources, energy, and labor, and produce products, services, and waste. With the deepening of urban metabolism research, it is possible to unify different forms of materials and energy and convert them into solar emergy joules (Sej) with the appropriate emergy transformity as forms of materials and energy are finally transformed into solar energy. Combined with social and economic data of the study area, it is possible to assess regional development through emergy analysis (Odum, 1996). According to the emergy theory suggested by Odum (1996), we collected the study area’s natural and social economic data for 2000∼2016, and then we applied emergy analysis to evaluate the internal and external energy flow between urbanization and environment. The calculation formula of the emergy of the studied area is:

(11) \begin{eqnarray*}E{m}_{T}=\sum E{m}_{i}=\sum {E}_{i}\mathrm{ \ast }T{r}_{i}\end{eqnarray*}

where *Em*_*T*_ represents the total energy value of the socio-economic-ecological complex ecosystem; *Em*_*i*_ represents the energy value of the *i* resource or product; *Tr*_*i*_ indicates the emergy conversion ratio of the *i* resource or product; and units of emergy is *sej*^−1^.

**Figure 2:**
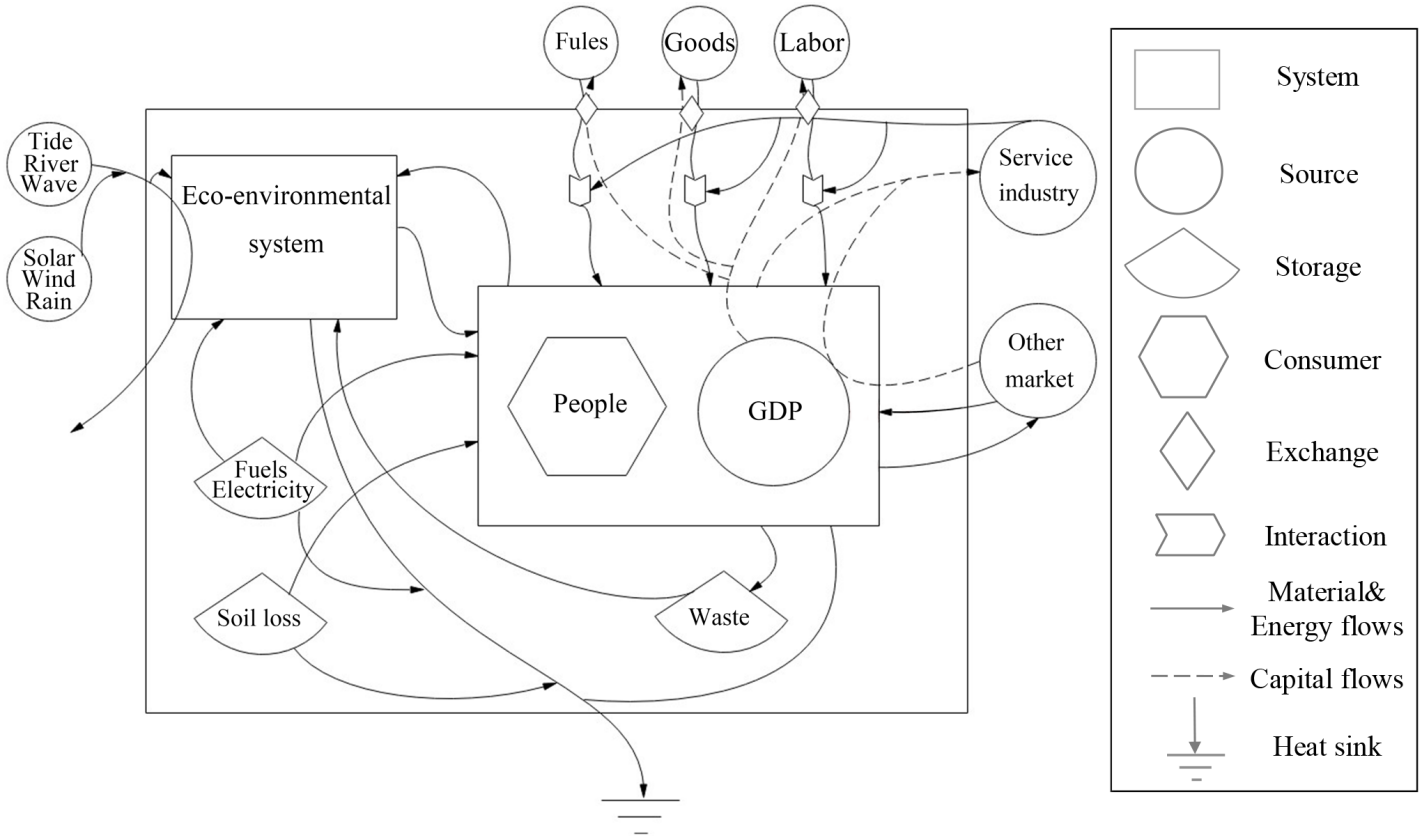
Emergy diagram of Dongying-Binzhou urban area. The arrow outside the system on the left represents resources that are not being utilized by the system. The arrow below the bottom of the system represents heat sink.

An emergy diagram in Fig. 2 shows the emergy flows of the socio-economic-ecosystem in the Dongying-Binzhou urban area.

The local renewable resources of the complex ecosystem include solar energy, rain potential and chemical energy, wind energy, wave energy, tidal energy, river potential and chemical energy, and earth cycle energy. Renewable resource products include agricultural products, livestock products, and aquatic products; non-renewable resources include topsoil loss and soil loss; non-renewable resources products include steel, natural gas, petroleum, export services and commodities; and waste includes solid waste and waste water.

The emergy of the socio-economic-ecological composite urban ecosystem analyzed in this study includes local renewable resources (R), rough non-renewable resources (N_0_), local centralized used resources (N_1_), import resources (IMP), export resources (EXP), and waste (W). Local renewable resources (R) include river potential energy, tide, and earth cycle. Rough non-renewable resources (N_0_) contain topsoil loss and soil loss. The local centralized used resources (N_1_) include natural gas, raw coal, gasoline, diesel, fuel oil, and electricity. Import resources (IMP) mainly comprise imported goods, foreign investment, and tourism income. Export resources (EXP) consist of export goods and investment output.

The metabolism between urbanization and environment coupled with the application of emergy analysis includes the following aspects: internal structural characteristics, metabolic intensity, environmental metabolic pressure and the level of sustainable development within the system, and environmental load changes from external factors. Combining the existing data, the seven subsystems of the socio-economic-ecological subsystem are selected for analysis and evaluation, including total emergy used (U), emergy per capita (U_cap_), emergy extroversion ratio (EER), emergy to money ratio (EMR), emergy self-support ratio (ESR), environmental loading ratio of internal elements (IELR), and environmental loading ratio of external elements (EELR) (Table 3).

**Table 3:** Subsystems of the socio-economic-ecological subsystem.

Item	Expression	Unit
Social subsystem emergy analysis		
1. Total emergy used (U)	R+N_0_+N_1_+IMP	Sej
2. Emergy per capita (U_cap_)	U/population	Sej/cap
Economic subsystem emergy analysis		
3. Emergy extroversion ratio (EER)	IMP+EXP/U+EXP	
4. Emergy to money ratio (EMR)	U/GDP	Sej/$
Natural subsystem emergy analysis		
5. Emergy self-support ratio	(R+N_0_+N_1_)/U	
6. Environmental loading ratio of internal elements (IELR)	(N_0_+N_1_)/R	
7. Environmental loading ratio of external elements (EELR)	IMP/R	

**Notes.**

a R: local renewable resources.

N_0_ rough non-renewable resources N_1_ local centralized used resources IMP import resources EXP export resources

### Data sources

The data used in this study mainly come from the Shandong Province Statistical Yearbook (Shandong Statistics Bureau, 2001–Shandong Statistics Bureau, 2017), as well as the China Statistical Year Book on Environment (National Bureau of Statistics; Ministry of Environmental Protection, 2001–National Bureau of Statistics; Ministry of Environmental Protection, 2017). The meteorological data used for emergy calculation are from the China Meteorological Data Service Center (2018).

## Results

### Calculation and analysis of coupling degree and coupling coordination degree

The coupling degree and coupling coordination degree between urbanization and environment in Dongying and Binzhou were calculated using formulas Eqs. (9) and (10). The calculation results are shown in Table 4 and Table 5.

**Table 4:** Coupling degree of the study area.

City/Year	2000	2002	2004	2006	2008	2010	2012	2014	2016
Dongying	0.806	0.901	0.969	0.979	0.995	0.999	0.992	0.980	0.974
Binzhou	0.965	0.978	0.998	0.999	0.999	0.998	0.999	0.995	0.993
Dongying and Binzhou	0.847	0.899	0.978	0.989	0.990	0.995	0.998	0.996	0.974

**Table 5:** Coordination degree of the study area.

City/Year	2000	2002	2004	2006	2008	2010	2012	2014	2016
Dongying	0.578	0.604	0.648	0.635	0.608	0.626	0.676	0.743	0.809
Binzhou	0.478	0.497	0.506	0.522	0.535	0.569	0.601	0.662	0.703
Dongying and Binzhou	0.557	0.589	0.611	0.632	0.659	0.721	0.756	0.803	0.821

By calculating the coupling degree and coupling coordination degree between the urbanization and environment of the study area, we found that the coupling degrees of Dongying and Binzhou tended to be stable and were larger than 0.8 from 2000 to 2016, suggesting that these areas were in well-coupled states, and rapid urbanization was enabled by the rich ecological resources. Meanwhile, the coupling coordination degree of Dongying from 2000 to 2016 steadily increased from 0.578 to 0.809, indicating relatively high-level coordinated coupling of urbanization and environment. At the same time, the coupling coordination degree of Binzhou increased from 0.478 to 0.703. It was moderately imbalanced from 2000 to 2003 and mildly coupled during 2004-2016. The coupling coordination degree of the two cities increased from 0.577 to 0.821 while the coordination rank was mildly coordinated from 2000 to 2013, suggesting that the protection of environment gained attention due to the rapid urbanization and increase in the investment in environmental protection. Furthermore, urbanization and environment were well-coordinated in the last three years (2014∼2016), suggesting that environmental protection was a priority in urban construction policies.

### Analysis of the social subsystem of total emergy and metabolic structure change

**Figure 3:**
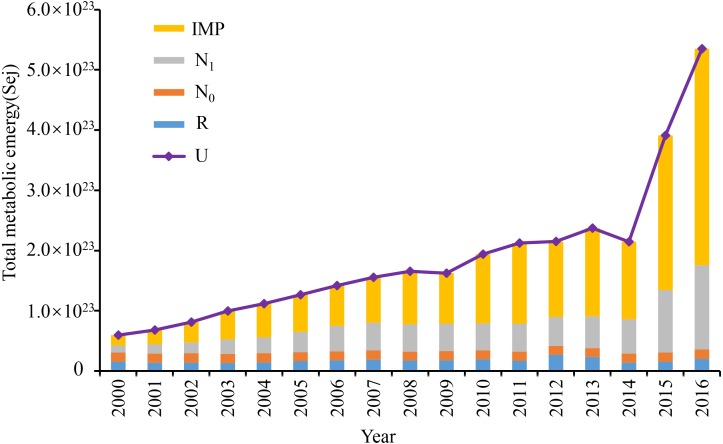
Total metabolic emergy of the Dongying-Binzhou urban area from 2000 to 2016. R, local renewable resources. N_0_, rough non-renewable resources. N_1_, local centralized used resources. IMP, import resources. U, total emergy used.

The results showed that the total emergy in Dongying and Binzhou exhibited an upward trend from 2000 to 2016. However, the rise of total emergy was significantly different during different stages between 2000 and 2016 (Fig. 3). The average energy growth rate was 13% between 2000 and 2008, 22.6% between 2009 and 2016, and 35.7% from 2014 to 2016 in particular. These results reflect the fact that urban emergy growth was relatively slow between 2000 and 2008. Dongying and Binzhou are energy-consuming cities and development was slow in this period. However, the total emergy increased rapidly between 2008 and 2016, suggesting that the development of Dongying-Binzhou urban area was affected by the rapid urbanization process at this stage. In particular, urbanization further accelerated from 2014 to 2016, driven by the “12th Five-Year Plan” policy from 2011 to 2016. The development of some other urban regions was markedly different. For example, the total emergy increased from 2. 4 ×10^23^ Sej to 4. 75 ×10^24^ Sej in the Beijing-Tianjin-Hebei agglomeration, with an average rate of increase of 32%, while the total emergy in Dongying and Binzhou increased from 5. 87 ×10^24^ Sej to 6. 44 ×10^24^ Sej with an average rate of increase of 3.14% (Fang & Ren, 2017).

With the acceleration of urbanization, the emergy metabolic structure of the Dongying-Binzhou urban area underwent substantial changes from 2000 to 2016, gradually shifting from the dominance of internal elements to that of internal and external multi-elements. However, data show that the proportion of locally centralized resources and external elements remained relatively high between 2000 and 2016. During this period, the emergy metabolic structure of the Dongying-Binzhou urban area was mainly composed of N and IMP. In contrast, the proportion of external emergy increased from 15.09% to 57.61% during 2000∼2016, gradually surpassing the proportion of internal resources (Table 6). Meanwhile, the proportion of internal centralized resources (N_1_) declined from 54% to 36% during 2000∼2016 (Fig. 3). The results indicate that the Dongying-Binzhou urban area is experiencing a transition from internal to external emergy dominance.

**Table 6:** The percentage of internal and external elements changes in the Dongying-Binzhou urban area from 2000 to 2016.

year		2000	2002	2004	2006	2008	2010	2012	2014	2016
Emergy percentage of internal elements (%)	Dongying	7.27	11.97	26.63	34.28	31.55	43.32	45.78	40.83	46.27
	Binzhou	29.77	41.95	49.80	47.33	53.31	59.41	58.26	59.90	67.16
	Dongying-Binzhou urban area	15.09	23.80	38.78	41.33	41.83	50.03	50.55	47.95	57.61
Emergy percentage of external elements (%)	Dongying	92.73	88.03	73.37	65.72	68.45	56.68	54.22	59.17	53.73
	Binzhou	70.23	58.05	50.20	52.67	46.69	40.59	41.74	40.10	32.84
	Dongying-Binzhou urban area	84.91	76.20	61.22	58.67	58.17	49.97	49.45	52.05	42.39

**Figure 4:**
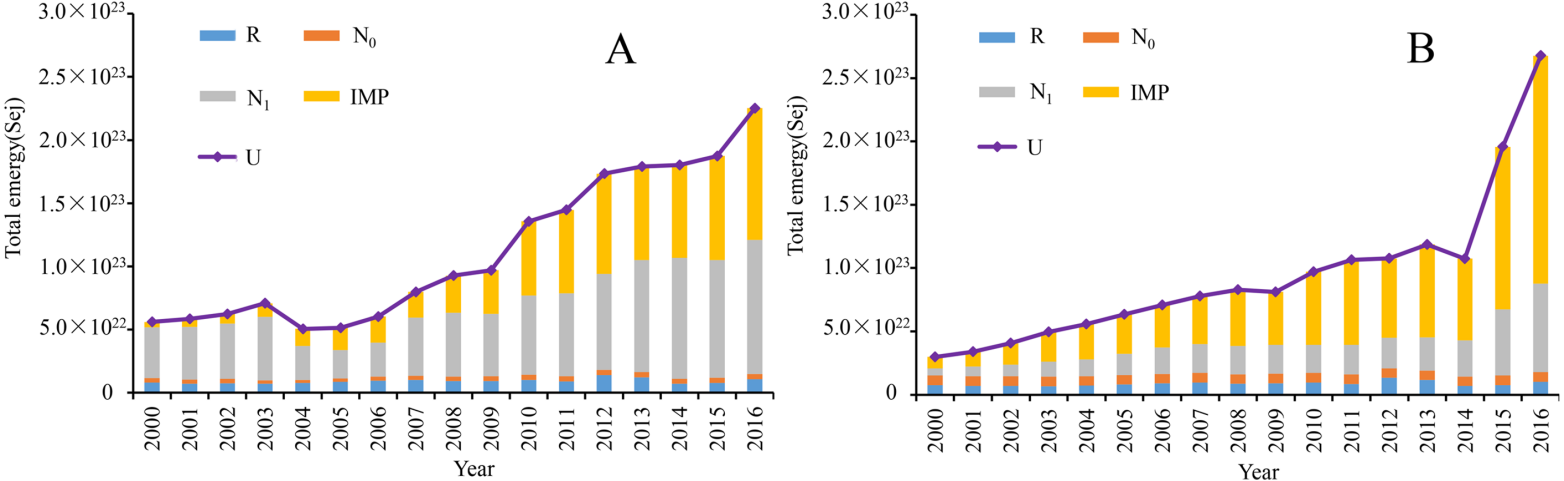
The total emergy of each of Dongying-Binzhou urban area from 2000 to 2016. (A) The total emergy of Dongying from 2000 to 2016 (B) The total emergy of Binzhou from 2000 to 2016. R, local renewable resources. N_0_, rough non-renewable resources. N_1_, local centralized used resources. IMP, import resources. U, total emergy used.

The metabolic emergy of Dongying and Binzhou was 5. 60 ×10^22^ Sej and 2. 98 ×10^22^ Sej in 2000, respectively, and reached 2. 25 ×10^23^ Sej and 2. 68 ×10^23^ Sej in 2016. The data showed that development of urban areas mainly depends on the supply of internal resources and the input of external resources, while the structure of internal emergy and external input emergy determined the direction and level of urban development. The emergy growth of Dongying was relatively slow during 2000∼2008 and increased significantly from 2009 to 2016, during which period the emergy of R and N_0_ was comparatively stable. While the proportion of N_1_ was much larger and the IMP grew more slowly from 2000 to 2008, the main metabolic emergy demands gradually transformed from internal emergy to external emergy, and the proportion of IMP increased rapidly from 2009 to 2016 (Fig. 4A). The total emergy of Binzhou increased relatively smoothly from 2000 to 2014 and grew rapidly in 2015 and 2016. The R and N_0_ hardly changed while the N_1_ maintained a small, steady increase from 2000 to 2013 and increased at a slightly faster rate from 2014 to 2016. However, the proportion of IMP had relatively stable growth between 2000 and 2013 and increased more rapidly in the last three years (from 2014 to 2016). The data showed that Dongying began to adjust its industrial structure and vigorously developed the tertiary industry after 2000, while taking steps to maintain the secondary industry, which was the main energy processing area in the Yellow Triangle. The proportion of external emergy in Dongying increased from 7.27% to 46.3% during 2000∼2016. These data reflected that the government focused on building the Yellow River Delta and began to vigorously develop the tertiary industry. During the reform and opening-up, the proportion of external resources increased significantly (Fig. 4B). In contrast, the proportion of external emergy in Binzhou increased from 29.8% to 67.2% from 2000 to 2016, and the proportion of external input increased significantly, accounting for more than 50% (Fig. 4B).

**Figure 5:**
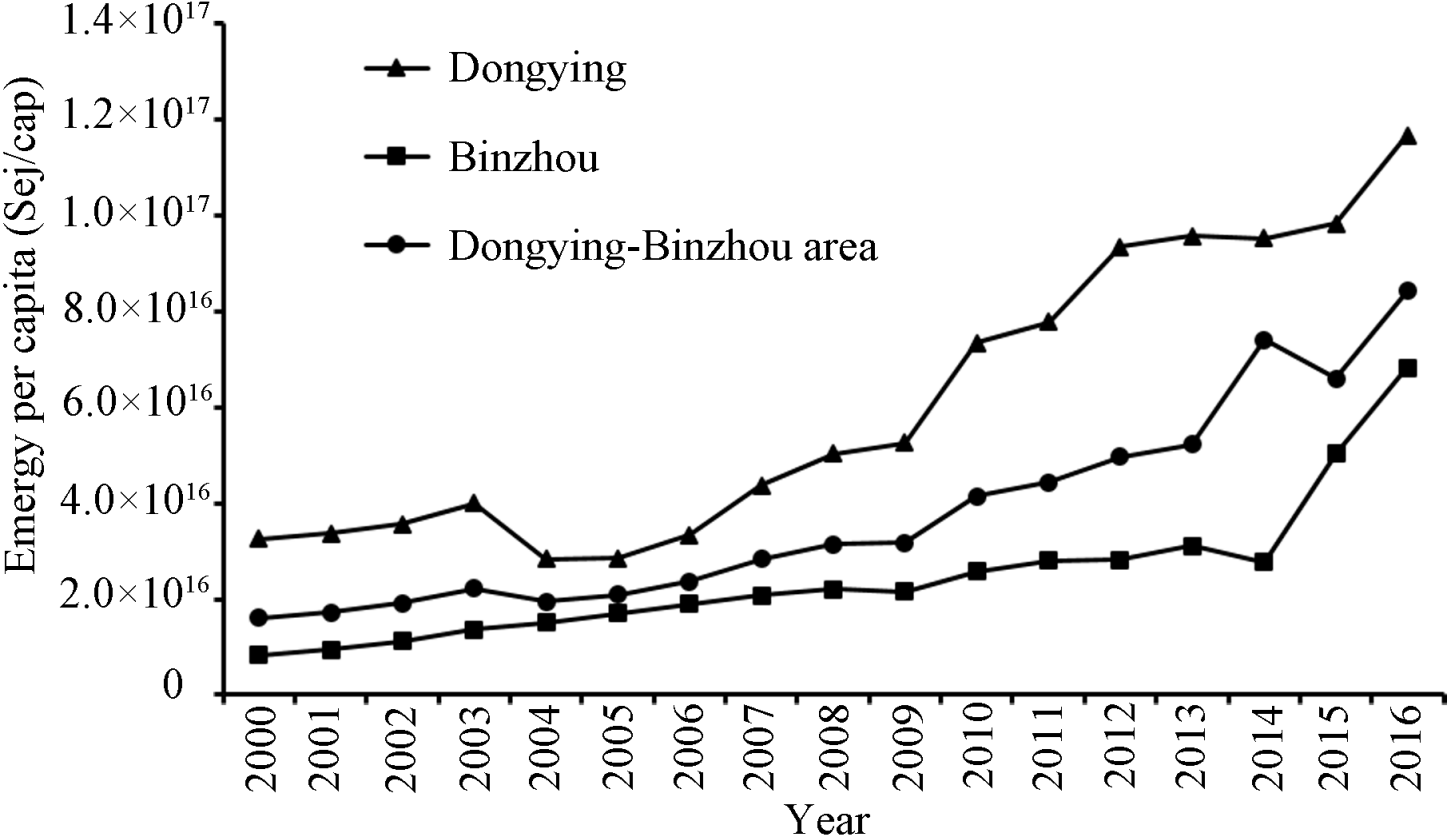
The emergy per capita of the Dongying-Binzhou urban area between 2000 and 2016. High emergy per capita represents a high living standards of urban residents.

The living standards of urban residents were measured by emergy per capita (U_cap_), which reflects living standards more accurately than the energy per capita. The results showed that the U_cap_ consumption of the Dongying-Binzhou urban area increased from 1. 61 ×10^16^Sej/cap to 8. 42 ×10^16^Sej/cap. They also showed that the U_cap_ of Dongying and Binzhou increased from 3. 25 ×10^16^Sej/cap and 8. 26 ×10^15^Sej/cap to 1. 17 ×10^17^ Sej/cap and 6. 82 ×10^16^ Sej/cap (Fig. 5), respectively. Data showed that the U_cap_ of Dongying is larger than that of Binzhou.

### Analysis of the economic subsystem of EER and EMR

**Figure 6:**
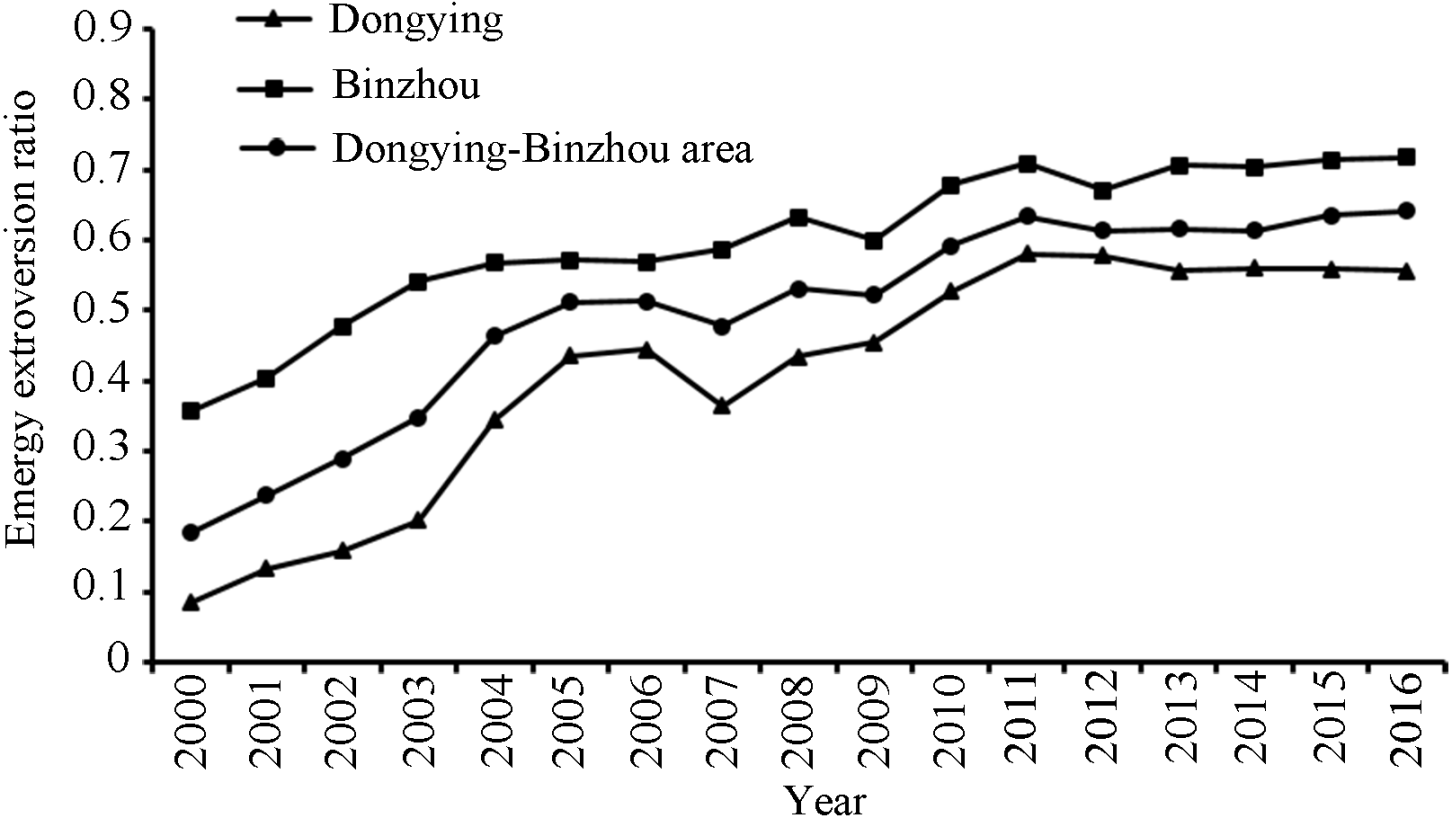
The emergy extroversion ratio of the Dongying-Binzhou urban area between 2000 and 2016. High emergy extroversion ratio represents a high degree of dependence on external factors.

As one of the most important indicators, the emergy extroversion ratio (EER) is used to reflect the dependence of external factors on the economic development of cities in the Dongying-Binzhou urban area. The external factors include both IMP and Export (Table 3). The growth patterns of Dongying and Binzhou have varying similarities and differences at every stage. The EER changes in Dongying and Binzhou can be divided into two phases. The first is the rapid growth phase from 2000 to 2006, during which the EER of Dongying and Binzhou increased from 0.084 and 0.356 in 2000 to 0.441 (or 5.25 times) and 0.568 (or 1.59 times) in 2006, respectively. However, from 2007 to 2016, the EER of both Dongying and Binzhou was in steady growth, increasing from 0.364 and 0.589 in 2007 to 0.555 and 0.716 in 2016, respectively, and the increase was relatively stable compared with the previous stage (Fig. 6).

**Figure 7:**
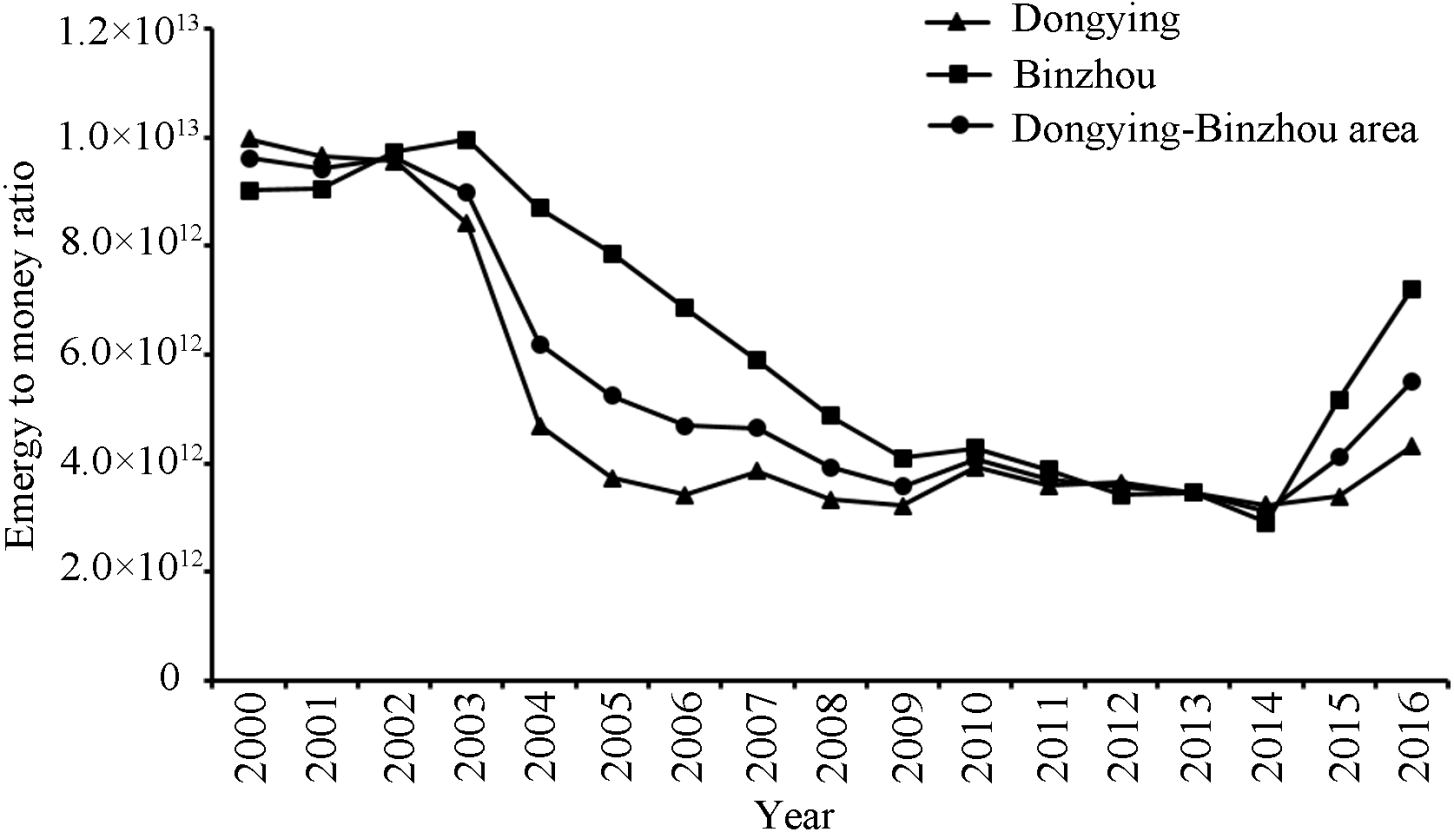
The emergy to money ratio of Dongying-Binzhou urban area between 2000 and 2016. High emergy to money ratio represents a low energy efficiency.

Emergy to money ratio (EMR) is used to quantify emergy use efficiency to characterize the metabolic intensity of elements inside and outside the regions. A high EMR generally represents a low energy efficiency. The results show that between 2000 and 2014, the metabolic intensity of internal and external factors in the Dongying-Binzhou urban area declined rapidly and the efficiency of emergy use gradually increased (Fig. 7). The EMR of the Dongying-Binzhou urban area decreased from 9. 62 ×10^12^ Sej/$ to 3. 10 ×10^12^ Sej/$. However, while the emergy intensity of Dongying and Binzhou increased, the utilization efficiency of emergy decreased. The EMR of the Dongying-Binzhou urban area increased from 3. 10 ×10^12^ Sej/$ to 5. 50 ×10^12^ Sej/$. Data show that the EMR of Dongying is higher than that of Binzhou, because Dongying actively carried out industrial restructuring, industrial technology upgrades, and rational allocation of resources, especially during the “Tenth Five-Year Plan” from 2001 to 2006. At the same time, Binzhou is mainly based on light and textile industries, meaning that the industrial structure was slow to adjust, and the technology update speed was relatively slower.

### Analysis of the ecological subsystem of ESR and ELR

**Figure 8:**
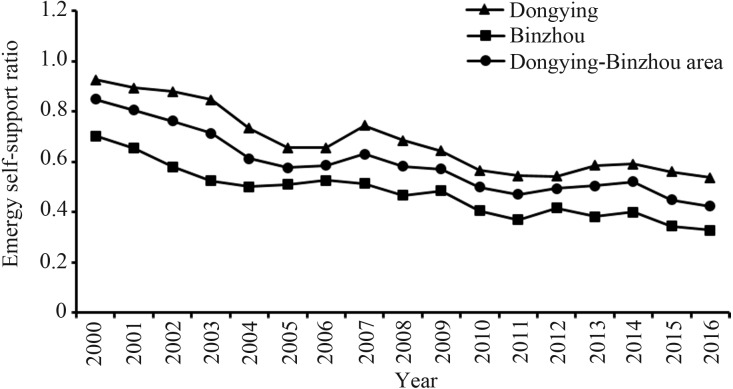
The emergy self-support ratio of Dongying-Binzhou urban area between 2000 and 2016. High emergy self-support ratio usually represents the relatively fragile environment.

Emergy self-support ratio (ESR) is the indicator that evaluates the self-sufficiency of the socio-economic-ecological system. This indicator can also reflect the supply capacity of the environment. A high ESR usually represents the relatively fragile environment. The results show that the ESR of the Dongying-Binzhou urban area dropped from 0.849 to 0.423 between 2000 to 2016, indicating that the metabolic emergy of the region is converted from the local internal supply to the supply of external elements (Fig. 8). Data show that the ESR of Dongying is relatively higher than that of Binzhou because a large part of the metabolic emergy of Dongying comes from internal elements.

**Figure 9:**
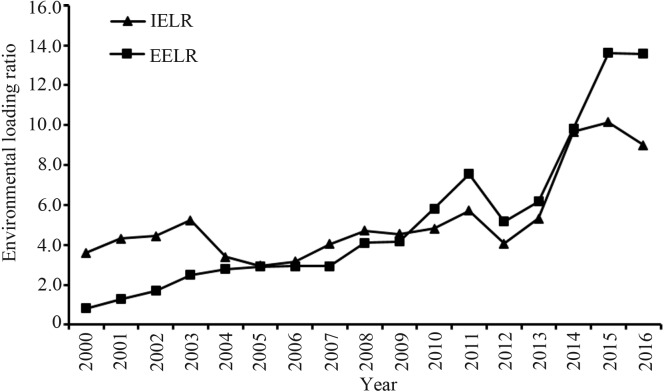
The environmental loading ratio of the Dongying-Binzhou urban area between 2000 and 2016. (IELR): The environmental loading ratio of internal elements. (EELR): The environmental loading ratio of external elements.

The traditional environmental loading ratio (ELR) refers to the level of environmental load (Bastianoni & Marchettini, 2000). However, this indicator cannot distinguish between the impact of internal elements and external import elements on the environment. The impact on the environment from internal and external aspects was discussed to explore the influence of different factors on the environment during the coupling process between urbanization and environment. The environmental loading ratio of internal elements(IELR) indicates the impact on the environment from local internal elements. Meanwhile, the environmental loading ratio of external elements (EELR) indicates the influence of external elements on the environment. Data show that both the IELR and EELR increased due to the combination of internal and external factors, and the environmental load caused by internal elements increased from 3.59 to 8.99 during 2000 ∼2016 (Fig. 9). Similarly, the environmental load caused by external elements rose from 0.82 to 13.58 during 2000∼2016, an increase of 16.70 times. The gap between internal and external elements also increased from 0.23 times to 1.51 times during 2000∼2016 as the sources of urban environmental pressure gradually shifted from internal to external factors.

**Figure 10:**
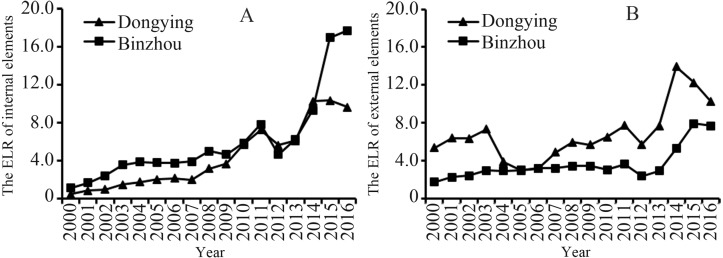
The environment loading ratio (ELR) of the internal and external elements of the Dongying-Binzhou urban area between 2000 and 2016. (A) The environment loading ratio (ELR) of the internal elements (B) The environment loading ratio (ELR) of the external elements.

The results show that the IELR of Dongying and Binzhou exhibited an overall upward trend from 2000 to 2016, and Dongying fluctuated drastically during this period. In Dongying, the IELR was 5.34 in 2000, dropped to 2.92 in 2005, peaked at 13.91 in 2014, and declined to 10.23 in 2016. In contrast, the IELR increased relatively slowly from 1.72 in 2000 to 3.61 in 2011, dropped to 2.36 in 2012, and increased rapidly to 7.67 from 2012 to 2106 (Fig. 10A). With the increase of external elements (EELR), their influence on the environment is becoming increasingly serious. In particular, Binzhou increased from 1.15 in 2000 to 17.7 in 2016, and Dongying increased from 0.49 in 2000 to 9.67 in 2016 (Fig. 10B).

## Discussion

### Relationship between urbanization and environment based on the coupling degree and coupling coordination degree

Our study on coupling degree analysis showed that urbanization and environment were comparatively well-coupled from 2000 to 2016 with a degree of 0.847–0.997.The degree for Beijing-Tianjin-Hebei region ranged from mildly coupled to well-coupled (0.712–0.826) (Wang, Ma & Zhao, 2014). In the Pan Yangtze River Delta region, the coupling degree increased from mildly coupled to well-coupled (0.549–0.974) during this period (Sun et al., 2017). From the trend of the coupling degree of these three areas, we can conclude that the interaction between urbanization and environment has gradually become complementary and inseparable. The coupling coordination degree was mildly coordinated and had an upward trend in the Dongying-Binzhou urban area from 2000 to 2016 (0.557–0.804), the interaction between urbanization and the environment was relatively good and the relationship was developing in a favorable direction ( Li et al., 2012). However, in some respects, urbanization and the environment were not well coordinated. By comparison, the coordination degree of the Beijing-Tianjin-Hebei region accelerated from mildly to well-coordinated in the same period (Wang, Fang & Wang, 2015). Compared with the Beijing-Tianjin-Hebei region, where plenty of external resources are always input, the improvement of the coupling coordination degree in the Dongying-Binzhou urban area depends on the transformation and upgrading of the production mode. Due to the construction strategy of the “Yellow River Delta High-tech Economic Zone”, measures were taken to increase the environmental protection investment in the process of urbanization to ensure that the environment can be coordinated with urban development. Although the pressure of external elements is increasing gradually during the cities’ transformation from resource-based to comprehensive cities, the sustainability of the environment could still be enhanced. Furthermore, the coupling coordination degree in the Pan Yangtze River Delta region ranged from 0.374 to 0.829 between 2000 and 2016; there is neither a large number of external resources nor a large number of mineral resources in this region, and the main mode of production is light industry (Sun et al., 2017). In the early stage of urbanization, environmental damage was quite significant. However, with the improvement of production technology and use of clean energy, the coordination between urbanization and environment improved. By comparing the development of the coordination degree of the three urban areas, we can conclude that attention should be paid to environmental protection in the process of urbanization and more policies should be proposed to reduce the environmental load. This will ensure the coordinated and sustainable development of urbanization and environment.

### Metabolic intensity and efficiency between urbanization and environment

The total metabolic emergy of the Dongying-Binzhou urban area increased during the analysis period. Compared with the Beijing-Tianjin-Hebei agglomeration (Fang & Ren, 2017), the average rate of this increase for the Dongying-Binzhou urban regions from 2000 to 2008 was much slower, but the rate of the Beijing-Tianjin-Hebei agglomeration declined from 2009 to 2016 due to the influence of national policies that controlled the total emergy consumption of social development. While the urbanization process of the Dongying-Binzhou urban area obviously lagged behind that of the Beijing-Tianjin-Hebei agglomeration, the emergy in Dongying and Binzhou increased at a rapid speed. Meanwhile, the regional structure had undergone a transition from being internal emergy dominant to external emergy dominant. In terms of individual cities, the EER of Dongying increased from 0.084 to 0.555 during 2000∼2016, while the external emergy of Binzhou rose from 0.356 to 0.716. It can be seen from the development trend of this period that Dongying transitioned from a development mode driven by the secondary industry to one driven by a combination of secondary and tertiary industries after the adjustment of the industrial structure. The current mode of production is mainly dependent on external resources and labor force to achieve the output of capital through processing, exhibiting the characteristics of an export-oriented urban metabolic model. In contrast, Binzhou, a city dominated by traditional light industry, actively carried out economic structural adjustment and industrial upgrading, vigorously developed the tertiary industry, and promoted increased interaction between urban areas and external elements. As a result, the metabolic pattern of export-oriented cities has begun to emerge. Meanwhile, with the entry of a large number of external energy values, the local demand for resources from the environment is reduced, which is conducive for the sustainable development of the environment.

The growth of U_cap_ suggests that as the structure of the industry continues to evolve, technology innovation continues, and economic efficiency continues to improve (Zhang et al., 2016), social welfare and living standards are improving. From previous results, it can be concluded that although the EMR of the two cities rose occasionally from 2014 to 2016, the overall EMR showed a downward trend, suggesting that the emergy utilization efficiency of the research area has continuously improved along with industrial technology upgrades and rational allocation of resources, and that environmental pollution and destruction have relatively decreased. The previous results of ESR showed a downward trend as well, indicating that local environmental pressure has been alleviated to promote sustainable development of the environment. Similarly, the EMR and ESR of the Beijing-Tianjin-Hebei region show the same trend (Cai et al., 2009; Fang, Liu & Li, 2016; Fang & Ren, 2017; Qi et al., 2017; Huang et al., 2018).

### Environmental load caused by internal and external elements on the environment in the process of urbanization

The environmental load from inside and outside the Dongying-Binzhou urban area has constantly increased in the last 17 years. The above data suggest that with the further reform and opening-up, the continuous expansion of cities and adjustment of industrial structures have led to the concentration of various production elements in the high-tech urban regions of the studied area. The gap between internal and external factors also increased from 0.23 in 2000 to 1.51 in 2016, and the sources of urban environmental pressure gradually shifted from internal to external aspects. The development of external factors such as capital, labor force, and tourism is a significant indicator of urban economic activities, which has a significant impact on urbanization. However, without proper policies, these factors are bound to have a negative impact on the development of society. In terms of individual cities, the IELR of Dongying fluctuated significantly between 2000 and 2016. The implementation of cleaner production resulted in a significant reduction in environmental load from 2000 to 2006, but from 2007 to 2013, the carrying capacity of the environment was limited due to rapid development of urbanization, and the environmental loading ratio rose rapidly. In 2016, the industrial structure was further adjusted, and policies to strengthen the protection of the environment were put forward to reduce the internal environmental loading ratio. In contrast, the IELR of Binzhou steadily decreased from 2000 to 2012; while in the last four years (2013∼2016), the consumption of internal elements increased due to urbanization, leading to the increase in IELR. With the increase of external import elements, the influence of external elements on the environment gradually becomes serious. Especially for Binzhou, where there is no pillar industry, the carrying capacity of the environment is facing a severe test due to the influx of external resources. At the same time, compared with the previous research on the Beijing-Tianjin-Hebei urban agglomeration, we find that the factors causing the environmental load in developing countries are gradually shifting from the inside to the outside in the process of transformation (Sun, Lu & University, 2014; Fang, Chen & Wang, 2015; Fang & Ren, 2017; Huang et al., 2018). For foreign cities, with the continuous adjustment of industrial structure, the use of clean energy and the implementation of more stringent emission reduction measures, the elements causing environmental load are also constantly changing. The environmental load caused by internal elements (local non-renewable resources, local mineral resources) in Roma, Italy has decreased by at least 50% in recent years, while the environmental load caused by external elements (imported fuel, electricity, and thermal resources) increased by nearly 30% (Viglia et al., 2017). Our results are consistent with these previous studies while provided new evidence for understanding the effects of urbanization on the environment.

Besides, we note that this study also has certain limitations that should be addressed in the future. The external elements have more effects in the resource-based cities at the present stage, whether there is such a trend exist in non-resource-based cities. With the increase of external environmental load, what influence will be generated on the urban system. At the same time, it is worth noting that constructed wetlands are on the rise, whether this will ease the urban pressure on ecosystems. Furthermore, the application of emergy analysis model has some limitations, for example, many results are calculated by empirical formulas, and the correlation coefficients of formulas will change with continuous research. To cope with these issues, in the future, it is necessary to combine the knowledge from different disciplines including urbanization, regional policy, management and economics.

## Conclusion

In this study, the coupling degree of urbanization and environment and the degree of coupling coordination were calculated in two resource-based cities, and the energy metabolism of the coupled urban areas was analyzed. The conclusions are summarized as follows:

(1) The coupling coordination degree of urbanization and environment has been greatly improved in the cities of Dongying and Binzhou.

(2) The total metabolic energy of the two cities increased from 2000 to 2016, and external elements began replacing internal elements as the dominant factor.

(3) The elemental metabolic intensity of these cities gradually decreased between 2000 and 2016. Over the same period, the emergy per capita increased significantly, suggesting the improvement of social energy utilization efficiency, social welfare, and living standards.

(4) Environmental load in the Dongying-Binzhou urban area continued to increase from 2000 to 2016, and the elements which result in environmental load were gradually transferred from the inside to the outside.

(5) The factors that cause environmental load in developing countries are gradually shifting from internal to external, which is vital to understanding of the global effects of the impact of urbanization on the environment.

In addition, it is necessary to combine knowledge from different disciplines, such as economics, management, policy and geography to further study the relationship between urbanization and environment.

##  Supplemental Information

10.7717/peerj.6869/supp-1 Supplemental Information 1 Emergy calculation raw data

## Additional Information and Declarations

### Data Availability

The following information was supplied regarding data availability: The raw data are available in File S1.
